# Potential Impacts of Prolonged Face Mask Use on Temporomandibular Joint Health as Neglected Lifestyle Repercussions of COVID-19 Pandemic—A Narrative Review

**DOI:** 10.3390/medicina60091468

**Published:** 2024-09-08

**Authors:** Szymon Jozef Pietrzyk, Emilia Kielczynska, Martyna Kowalczyk, Mateusz Mazurek, Zygmunt Antoni Domagala

**Affiliations:** 1 Clinical and Dissecting Anatomy Students’ Scientific Club, Wroclaw Medical University, 50-368 Wroclaw, Poland; emilia.kielczynska@student.umw.edu.pl (E.K.); martyna.kowalczyk@student.umw.edu.pl (M.K.); mateusz.mazurek@student.umw.edu.pl (M.M.); 2 Division of Anatomy, Department of Human Morphology and Embryology, Wroclaw Medical University, 50-368 Wroclaw, Poland; zygmunt.domagala@umw.edu.pl

**Keywords:** temporomandibular joint disorder, TMD, SARS-CoV-2 outbreak, COVID-19, COVID-19 pandemic, face masks, prolonged face mask use, TMJ, complications

## Abstract

Since December 2019, COVID-19 has rapidly spread worldwide, prompting the World Health Organization (WHO) to declare it a pandemic and advocate for the widespread use of face masks to mitigate transmission. In this review, we delve into the potential impact of prolonged face mask use on temporomandibular joint (TMJ) health, an area that has garnered limited attention amidst COVID-19 research. Research has revealed that improper mask fit and constant readjustment can lead to TMJ abnormalities. Similarly, there is a demonstrated correlation between continuous mask usage and an increased incidence of headaches, temporomandibular pain, and diminished quality of life. Many studies have highlighted discomfort in the preauricular area, headaches, TMJ noises, headache, jaw pain, and muscle fatigue, as well as dermatological disorders, which have been attributed to prolonged mask wear and its impact on TMJ health. Our study catalyzes future research endeavors, urging a deeper exploration of the implications of long-term mask wear, not only in the context of the COVID-19 pandemic but also among occupational groups regularly exposed to extended mask use. By unraveling the complexities of TMJ health in the face of evolving preventive measures, we aim to enhance our understanding of this issue and safeguard the well-being of mask-wearers worldwide.

## 1. Introduction

COVID-19 is an infectious disease caused by SARS-CoV-2 transmitted by droplets and close contact. The first human cases were reported in December 2019 in Wuhan City, China [1]. Most infected people experience mild to moderate respiratory illness and recover without special treatment. The risk groups with a higher chance of developing a serious illness are people with cardiovascular diseases, chronic respiratory diseases, cancer, the elderly, and diabetics. Anyone can get sick with COVID-19 and become seriously ill and die at any age [2]. Among many lifestyle changes the pandemic brought, like sleep, work, and shopping changes, were social distancing and masking [3,4,5,6,7,8,9,10].

In April 2020, when COVID-19 began, the WHO issued a statement that it did not recommend the wide use of masks by healthy people in the community [11]. Later, in June 2020, the WHO updated its advice on preventing COVID-19 when transmission from presymptomatic or even asymptomatic individuals was self-evident. This guidance targeted the continuous use of medical masks by health workers in clinical areas and included recommendations for mask use in the general population [11]. Instructions were based on results of observational studies that found out that wearing a mask is associated with a reduced risk of infection among health workers [12]. Masks aim to prevent the spread of the virus through direct contact and to protect the wearer from infection [13].

Masks have been recommended as a strategy of prevention to suppress transmission and save lives. Face masks are used to trap the particles or fluid drops in a filter to avoid or reduce the transfer of particles between the human lungs and the external environment [14]. The most used types of protective masks during the pandemic were surgical, N95, cloth, and FFP2/FFP3. N95 masks offer higher degrees of protection than surgical and fabric masks, but most N95 respirators fail to fit the face adequately. Fit is critical to provide promised protection for the wearer [15]. To ensure the effectiveness of masks, rules such as physical distancing, keeping rooms well ventilated, personal hygiene, and avoiding mass gatherings need to be followed.

The use of face masks was associated with the exacerbation of acne and the appearance of new acne lesions, as well as the deterioration of the general condition of the skin [16,17]. These adverse reactions may result in scratching and touching the mask, which reduces its effectiveness in protecting against virus transmission [18].

The continuous use of masks by medical personnel causes headaches de novo, exacerbates the pain, and increases its frequency in susceptible people [19,20,21,22]. One of the presumed causes may be respiratory alkalosis and hypocarbia. It was noted that most health workers had to take analgesics. Some even took time off work for this reason [23]. A survey of operating room staff found that N95 masks cause more discomfort than surgical masks. This is probably due to the tightness of this type of mask [24]. Pain related to protective mask wearing can be caused by pressure on a nerve or its branch, and it increases the longer the mask is worn. Pain was localized in the nasal region, followed by pain in the zygomatic, parathyroid, temporal, occipital, and submental regions. All were areas of contact with face masks. The localization of most symptoms suggests the involvement of the trigeminal nerve, particularly the maxillary and mandibular branches, and the localization of the pain can usually be attributed to the involvement of single, small, or terminal trigeminal branches. Fifteen percent of the population complaining of neuropathic pain also reported suffering from reduced skin sensitivity, probably because of skin inflammation involving the intradermic terminal nerve fibers [25].

The TMJ is a paired articulation that consists of two surfaces located on the mandible and the squamous part of the temporal bone. This joint enables symmetrical movements—protrusion and retraction of the mandible, raising and lowering the mandible—and asymmetric chewing movements. The TMJ is the only movable joint in the skull. Improper adhesion of the articular surfaces causes joint dysfunction, which is manifested by an incorrect moving path of the mandible and characteristic crackling sounds [26].

To provide protection against the virus, the mask must fit tightly to the nasolabial region and the TMJ [11]. We suspected that the previously mentioned pressure on the nerves caused by the mask might directly affect the TMJ [25]. Constant tension and abnormal positioning could lead to temporomandibular joint disorders (TMDs) [27,28].

TMDs are a broad term referring to all neuromuscular and musculoskeletal conditions of the masticatory muscles, TMJ, and the adjacent structures. The etiology of TMD is complicated, and it is likely multifactorial, with biomechanical, neuromuscular, psychosocial, and biological influences. Possible causes involve trauma, parafunctional habits, occlusal overloading, increased joint friction, depression, stress, anxiety, and alexithymia [29]. The symptoms of TMDs could be arthralgia, masticatory spasm, tightness around the face in the morning, otalgia, inability to fully open the mouth [30], and tension headaches, which may lead to depression [31]. Around 80% of patients treated for TMDs are women. The reason behind the sexual disparity is not clear, but both animal and human studies have suggested elevated levels of estrogen may be a predisposing factor. Peak TMD occurrence appears between the second and fourth decade of life [32,33].

During the COVID-19 pandemic, masks generated a sense of security, though not many treatment options were available [34]. In 2020, daily protective mask-wearing became the new normality [25]. The effects of wearing face masks during the pandemic on headaches and discomfort are well fathomable. The mandatory use of face masks, despite the side effects, was inevitable to arrest the coronavirus disease 2019.

The study aimed to explore the influence of masks on the TMJ. We hypothesized there is no link between face mask use and TMD. The duration of mask-wearing does not increase discomfort in the temporomandibular region.

## 2. Materials and Methods

Given the niche character of our topic, we decided to make our search as broad as possible. In our research, we used 5 databases: PubMed, Embase, Ebsco, Web of Science, and Google Scholar. The search took place from 10 to 16 February 2024. We did not register our research in any register of systematic reviews, such as PROSPERO. We searched for studies from the oldest to 10th of February. We did not set limitations on the type of papers; however, we focused on original articles.

Our search focused on abnormalities related to the TMJ that may have been caused by the prolonged use of a face mask as a self-protection measure. We used a combination of keywords where only one combination was used per search. The structure of the algorithm for making the search combinations was as presented below:

((“face mask” AND (“temporomandibular joint” OR TMJ))) AND (“degeneration” OR “pathology” OR “injury” OR “influence” OR “pain” OR “discomfort” OR “stiffness” OR “TMD” OR “arthralgia”).

To check the search strategy for every database see Supplementary Material S1.

In the next step, two authors (E.K. and M.K.) performed a thorough search of all chosen databases using this algorithm. The search was set for all fields. Focusing only on original articles, titles and abstracts of the papers were analyzed. After that, one of the authors (S.J.P.) checked for duplicates and reviewed the relevance of the studies. Studies on the influence of face masks on the TMJ were accepted for further analysis. Any disagreements or uncertainties where consulted with one other author (M.M.).

Later, in-depth analysis was performed by one researcher (M.K.) and included the use of JBI critical appraisal tools. Its outcomes are depicted in Table 1 and Table 2. We provide filled checklists in a PDF form (Supplementary Material S2–S5). This work was reviewed by two other authors (S.J.P. and E.K.). Then, the outcome of each paper was presented independently, focusing mainly on the effects of masks on the TMJ; nonetheless, other influences that were found in the studies were also pointed out.

## 3. Results

The initial search yielded *n* = 21 studies (PubMed *n* = 4, Embase *n* = 5, Ebsco *n* = 4, Web of Science *n* = 4, Google Scholar *n* = 4). After excluding duplicates (*n* = 16), we screened these papers and excluded one irrelevant study (which did not show a direct influence of face mask use on the TMJ) after reviewing the full text. After that, we were left with *n* = 4 articles.

**Figure 1:**
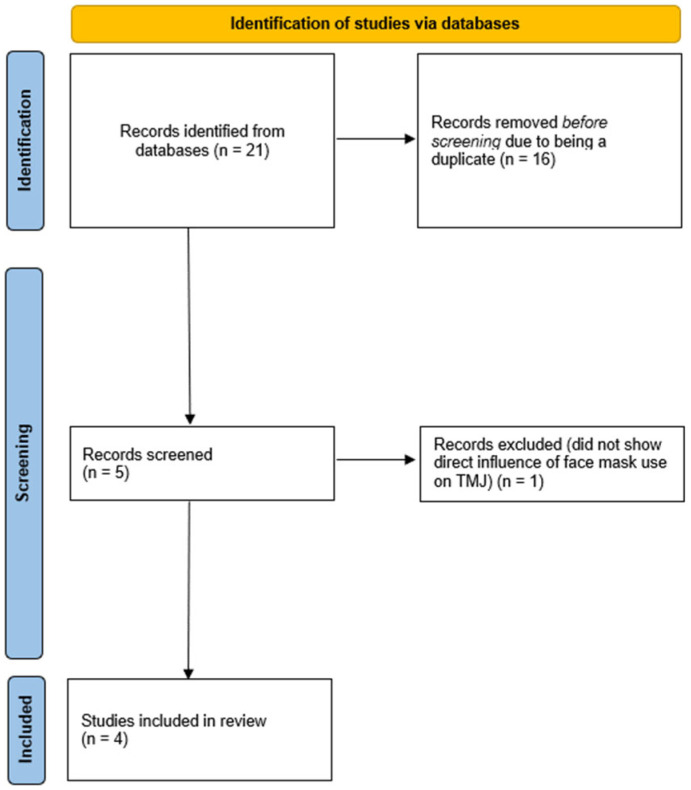
Flowchart depicting the process of systematization.

The process of systematization was depicted is a flowchart (Figure 1).

A total of four studies (Table 3) were used to confirm the negative influence of prolonged mask use on the temporomandibular joint [35,36,37,38]. We extracted some basic data from those studies and grouped them in Table 3. Other than basic metadata such as first author names, we gathered data on the studies themselves. We collected data on group size, country that the authors are affiliated with, and method of research. Moreover, in Table 3, we list the specific aims of the original studies. Those aims were set by the authors of the original studies directly or indirectly via the questions in the surveys they used to examine participants. The last column in Table 3 lists the main symptoms reported by the participants in the included studies.

Zuhour et al. [35] showed via a brief survey questionnaire that both the placement and the readjusting of masks have negative effects on the TMJ. The position of the mask depends on the dimensions of the face, mask elasticity, and its binding strength. Correcting such placement leads to mandible protrusion and rotation.

Patients who applied to the plastic surgery clinic with TMD complaints such as pain, articular noises, stiffness, joint lock, and joint tension were questioned on their mask usage and lifestyle habits. There were three groups:

- (39)—repetitive movements associated with masks as the only risk factor (study group);
- (55)—lifestyle/parafunctional habits;
- (12)—no known risk factors.

No participant changed their mask type of choice throughout the study. A total of 148 people participated in the study, but 42 were excluded because of comorbid diseases, TMD lasting more than a year (which misses the impact of COVID-19 on the TMJ), or any history of orofacial surgeries. Thus, 106 people in total participated in the survey. Questions about teeth grinding, gum chewing, teeth clenching, nail biting, and lip biting were included in the questionnaire. Various symptoms were reported, such as pain of the TMJ, articular noises, stiffness, joint lock, and joint tension; among the groups, the most common symptoms were the first two and the last one. The duration of mask wear varied among the participants; the majority wore them for 3–8 h per day in all groups. Cloth and respiratory masks were used, but surgical ones were most common in all three groups (a—60.2%, b—52.7%, c—49.8%).

Abnormal disk morphologic features were observed in the MRI of the patients. Most commonly thickening of the lateral pterygoid muscle was found. Two different radiologists evaluated the results based on blinded interpretations. Disk displacement in closed mouth position and reduction in open mouth was significantly higher in patients with mask-related repetitive movements (54.6%) than in those with parafunctional habits (33.3%) and with no known factors (30.4%). There was no posterior disk displacement reported.

TMJ action includes both forward and downward movements. Such repetitive motions are the cause of the destabilization of the ligaments supporting the joint. Incorrect mask sizing increases the negative outcomes of wearing masks. Differences in mask structure were the main limitation of the study.

Marques-Sule et al. [36] in their retrospective study during the COVID-19 pandemic touched not only on the effects of masks due to their mandatory continuous use on temporomandibular pain but also on headaches and the quality of life (QoL), the latter also being influenced by mask type.

People with musculoskeletal injuries in the craniofacial area, those who wore masks for a shorter period than 2 h (87 people), and institutionalized subjects were excluded from the study. A total of 542 participants were assessed for eligibility. The mean age of the participants was 37.89 ± 15.12. A total of 64.7% were women.

An online questionnaire was conducted, and questions included the appearance of bruxism, chewing discomfort, and TMJ pain. Symptoms are summed up in Table 4. Subjects were over 18 years old and wore face masks for at least 2 h a day. Face mask types used by subjects were as follows:

1. Cloth—63;
2. Surgical—328;
3. FFP2—151.

Headache was reported by 6.3% of the participants, and it was more intense (37.3%) with continuous mask usage (CMU) than without masks (31%). Participants were asked about the frequency, onset, description, intensity, duration, remission, location, and time of onset of their headache. The frequency of headache was most significantly influenced by cloth masks, then by FFP2, but when it came to surgical masks, there was no difference between using masks and not using them. A total of 77.7% of participants presented a gradual onset of headache, mostly reported as “once a week” (63.4%).

Headache impact was measured by the Headache Impact Test-6 (HIT-6). It provides information about the impact of the headache on a person’s performance and cognitive functioning, with answer scores as follows: never—6 points, hardly ever—8 points, sometimes—10 points, very often—11 points, and always—13 points. Forty-nine points or fewer are considered “little to no impact”. The mean score in the study was 45.56 for participants without masks and 47.46 with continuous mask use.

When TMJ discomfort was reported, it increased by 31% with FFP2 masks but not when it came to cloth or surgical masks. TMJ pain was characterized using a visual analog scale (VAS), with 0 meaning “no pain” and 10 meaning “maximum pain”. The mean score was 1.56 before mask use and 2.28 during mask use. TMJ pain reported while wearing face masks was differentiated between mask types: cloth (1.47), surgical (2.34), and FPP2 (2.10).

QoL was measured by Cantril’s Ladder of Life scale using an image of a ladder numbered 0–10, 0 meaning the worst possible life and 10 being the best possible life. Participants were asked two questions: “In which step do you think you are on without using a mask?” and “In which step do you think you are on using a mask?” QoL decreased regardless of mask type, meaning a 38% decrease for surgical masks and 31% for either FFP2 or cloth masks. The mean QoL score during mask use was 5.37 and 8.26 before mask use.

The problem of mask influence was also considered by d’Apuzzo et al. [37] via an observational cross-sectional survey using an online questionnaire that reached 665 people over the age of 18 years old. Two replies were excluded because of incomplete demographic documentation. Subjects from the general population taking part in the study wore masks as their usual routine during the pandemic. Pain in the preauricular area, noise at the TMJ, headache, and wearing modalities concerning masks were researched. The survey questionnaire was reviewed by three orthodontists and an expert in survey research from the University of Campania Luigi Vanvitelli in Napoli.

Participants were recruited using sponsored posts online. Of the subjects, 37% were health care professionals, and 21.2% of them were dentists. FFP2/FFP3 masks were used by 52.3% of the subjects, while 43.5% used surgical masks and 4.2% wore other types. Masks with two elastics were worn by 87% of the respondents, while masks with one elastic behind the neck were worn by 6.2%; also, 6.7% wore them with two elastics vertically on the head. The duration of mask wear differed among participants. Most (53.6%) wore masks for less than 4 h, 35.4% for 4–8 h, and 10.7% for more than 8 h a day, while 1.8% reported wearing them for 12 h consecutively.

In total, 400 participants reported new pain in the preauricular region while wearing masks, 263 reported no painful symptoms, and 147 participants felt pain during mask wear lasting for 4–8 h consecutively. The outcome differed between mask types: 41.3% of subjects using surgical masks reported pain/discomfort in the region anterior to the ears, while 54.3% of FFP2/FFP3 users reported the same.

There was no significant association between pain and consecutive time using the mask (*p* = 0.17), the kind of mask used (*p* = 0.32), or the mask-wearing mode (*p* = 0.26).

The duration of mask use in association with pain in the preauricular area is summed up in Table 5. The correlation between talking and pain in the region anterior to the ears is summarized in Table 6.

As shown above, most participants experienced pain in the preauricular area while wearing masks for at least 4 h consecutively.

Most people (92.2%) did not report hearing any noise in the TMJ region while chewing since wearing masks. In conclusion, no significant association was found with consecutive wear of face masks (*p* = 0.085). Seventy percent of subjects referred to avoiding talking while wearing masks because it increased the appearance of preauricular pain and new/increased noise heard at the TMJ.

When it came to headaches, 253 reported suffering from headaches during mask wear, while 411 did not report this symptom. Headaches were most common among subjects who wore masks for more than 8 h in total and for less than 4 h consecutively. This pain was most significant while wearing masks with two elastics behind the ears (87.4%) and with FFP2/FFP3 masks (57.7%).

The most negative effects were felt by users of FFP2/FFP3 masks who wore them for more than four hours continuously. Also, two elastics behind the ears increased the described influence.

Carikci et al. [38] investigated the effects of mask wear during the COVID-19 pandemic via a web-based cross-sectional survey. The influence of masks on discomfort, TMD, fatigue, and headache was researched.

People with a history of head or face surgery or a history of malignancy were excluded from the research. A total of 909 participants from the general population took part in the study. Participants differed from each other because of their occupation and working style: 384 of them were officers, 393 worked in the private sector, 15 were housewives, 21 were retired, 100 were students, and 16 were unemployed. Additionally, 651 worked in an office and 258 worked from home. Among the survey participants, three groups were formed depending on the duration of mask wear:

4. 1–4 h;
5. 5–8 h;
6. >8 h.

Mask type, apparatus, double masking, and daily mask changes among the participants are summed up in Table 7.

Jaw (14%), facial (16.1%), and neck (16.9%) pain, as well as cheek tension (16.1%), were the most intense in the third group, as was headache (51.9%). Different types of headaches were also distinguished between people. The most common headache types were temporal headache in Group 1 (25.7%) and Group 2 (33.7%) and tension headache in Group 3 (33.1%)

Pain at the trigger points of the TMJ, on M. Temporalis and M. Masseter, was measured by a Numerical Rating Scale (NRS) from 0 to 10, 10 being the worst pain. Both at the temporal and masseter muscle trigger points, pain was stronger in the second and third groups. TMD severity was evaluated by the Fonseca Anamnestic Index (FAI) and scored between 0 and 100. Scores of 0–15 meant no symptoms, 20–45 meant “mild”, 50–65 “moderate”, and 70–100 “severe”. The median score turned out to be 25 in Group 1 and 35 in Group 2 and Group 3. The duration of mask wear increased TMD prevalence and total fatigue. Masks could put pressure on the mentioned points, causing incorrect muscle movement and asymmetry of the system.

**Figure 2:**
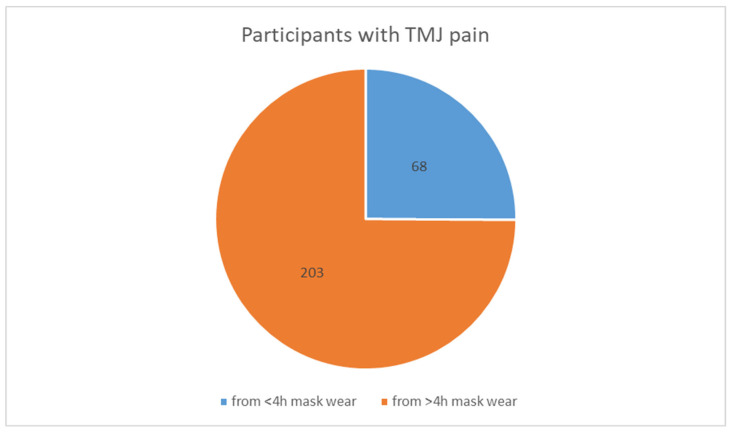
(by M.K.) Correlation between durations of mask use and TMJ pain in the studies by d’Apuzzo et al. [37] and Carikci et al. [38] (these data could only be summarized from these studies).

**Figure 3:**
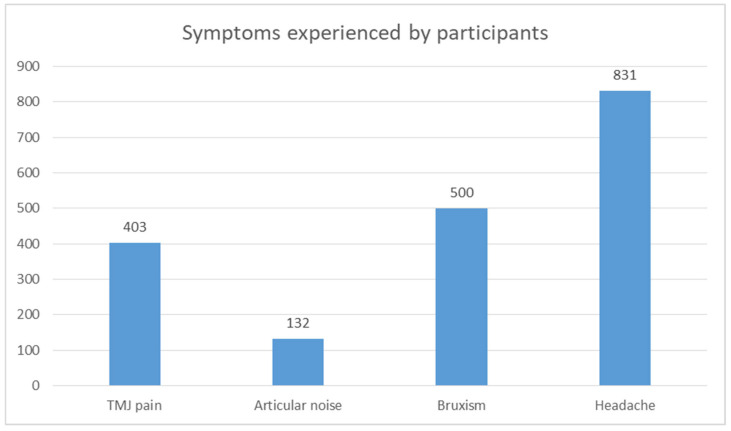
All reported symptoms.

A simple synthesis of the symptoms reported in the studies is presented in the tables below. Table 8, Table 9 and Table 10 summarize symptoms reported in two or more studies. TMJ pain was reported in all four studies found. A correlation between the duration of mask use and the prevalence of TMJ pain is shown in Table 11, and it is illustrated in the pie chart in Figure 2. Data regarding headache occurrence are gathered in Table 12. Figure 3 presents a chart with all symptoms reported that could be synthesized.

## 4. Discussion

Face mask use is not indifferent to the health and well-being of a person. As presented in the Introduction, masks can induce headaches, possibly due to alkalosis [23]. The studies we have found also discuss this matter, and their outcomes are no different [36,37,38]. Of the 2116 people included in those studies, 1364 participants reported headaches due to face mask use. There are also multiple reports regarding mask-induced dermatological problems. Acne is the most prevalent problem associated with mask use [16,17,39,40,41,42]; mask use can also reduce the general condition of the skin [17,43] or induce itch [18,44,45], which contributes to the overall discomfort that exists during mask use [24,46,47]. Face masks can increase transepidermal water loss, skin hydration, erythema, pH, and sebum secretion, which all can contribute to worse skin condition [48]. One review stated that the microclimate under the mask can be like a greenhouse, which favors the microbiota involved in the development of acne [49]. One of the biggest causes of discomfort are also breathing problems [42,50,51,52,53], especially in people suffering from asthma [53]. The prolonged use of face masks can also affect ocular health by exacerbating dry eye symptoms [54,55,56,57] or creating visual artifacts due to improper fit [58], which, as one of the existing cases showed, also plays a role in worsening those symptoms by directing the exhaled air into the user’s eyes [59]. It is worth noting that one of the studies discussed in this paper regarding the influence of mask use on TMJ disorders also showed that the improper fit of a face mask can impair this joint’s health due to repetitive movements [35], which could be induced by discomfort coming from blowing exhaled air into the eyes.

Mask use can also have a toll on the doctor–patient relationship, as it affects the effectiveness of clinical practice by altering the ability to understand the words that the other person is saying [60]. This is more visible in people with impaired hearing [61,62]. Alarmingly, masks decrease CFMT-K (Cambridge Face Memory Test for children [63]) scores, which means they also influence face recognition in children [64]; furthermore, masks decrease the quality of communication between children and teachers who wear masks, which can be caused by insufficient volume of the mask user’s voice and lack of information from lip movements [65]. Among other psychological aspects, masks induce anxiety in people who use them for longer periods [66,67].

The number of studies present regarding the influence of face mask use on TMJ health is very limited. We identified only four papers published in peer-reviewed journals. Even though we searched for papers discussing disorders correlated with prolonged mask use from any year, the papers that fit our criteria discussed only patients that had TMDs due to face mask use during the COVID-19 pandemic. We are aware of the psychological effect that it had on people all over the world and its importance in developing TMDs and bruxism due to elevated stress and anxiety (to which masks also can contribute, as stated in the previous paragraph), which was discussed in two systematic reviews [68,69]. This shows the importance of expanding the research to different professions with prolonged face mask use (i.e., surgeons, operating room staff, pathologists), as COVID-19 could have influenced the outcome of the studies solely based on pandemic patients. These were the main limitations of this study.

From existing research, it is apparent that the prolonged use of face masks can be associated with TMJ discomfort [36]. Research also suggests face mask use can even cause pain in the preauricular area [37], more prominent with repetitive jaw movements [35], which is one of the diagnostic criteria for temporomandibular disorders [70,71]. One of the studies [38] suggested that the prevalence of temporomandibular disorders can be linked to pressure put by the mask on the muscles responsible for masseteric movements. There is evidence that this may be one of the mechanisms, as wearing a mask influences the resting bioelectric activity of masticatory muscles, which was observed in healthy patients [72] and patients suffering from TMD [73]. According to Florjanski et al. 2019 [74], biofeedback is a useful tool in working with masticatory muscle activity, which elevates the trustworthiness of these studies.

One of studies found also implies that wearing an incorrectly sized mask can be a major factor in the development of TMJ dysfunction symptoms [35]. Improper face mask fit forces the user to adjust it by moving the mandible which, when repeated chronically, favors the development of TMD symptoms (86,6% for TMJ pain, 82,1% for articular noises, and 64,6% for joint tension in the group where repetitive movements associated with masks occurred as the only risk factor). When it comes to the type of mask, FFP2 and FFP3 masks have a higher risk of causing discomfort or pain in the temporomandibular joint [37,38] in comparison to surgical or cloth masks due to increased pressure put on the user’s face. The use of FFP3 masks over the recommended 1 h can even lead to facial pressure injuries [75]. It creates a possibility that if the pressure put on the user’s face is significant enough to cause an injury as an effect of prolonged use, it could also influence the position of the mandible and thus the health of the temporomandibular joint. When searching for a substitute, it is safer to lean towards surgical masks, as cloth/fabric masks have been proven to have lower protective quality [76,77]. There is also a potential to enhance surgical masks by infusing them with quaternary ammonium salts [78], which makes them an even better substitute for the FFP3 type. Devices like the one proposed by Bao et al. 2021 [79] can decrease the time of mask usage while simultaneously providing a safe environment for proximity settings.

Despite our wide range of research, we could not identify any more studies on this topic. We think it is a missed opportunity that there are so few studies that would explore this problem because in our opinion there is potential in connecting prolonged mask use with TMJ abnormalities when working with TMD patients. Adding prolonged mask use as a risk factor for developing abnormalities in the temporomandibular joint into the diagnostic process could improve the rate of correct diagnoses, especially after the COVID-19 era, and in people whose job is strictly connected with long periods of mask use (i.e., surgeons, anesthesiologists, surgical nurses, and other members of operation block staff, as well as pathologists).

If we look at the definition of quality of life (QoL) established by the World Health Organization (WHO), it is defined as “individuals’ perceptions of their position in life in the context of the culture and value systems in which they live and about their goals, expectations, standards and concerns” [80]. We can easily conclude that all of the existing problems that masks are responsible for can affect an individual’s QoL. This includes the topic we discuss in this paper—the temporomandibular joint. What is more, one of the studies included in this review showed a decrease in QoL with the use of all mask types [36], measured by two Cantril’s Ladder of Life scales which compared the well-being of participants who did and did not use masks.

## 5. Conclusions

Considering our findings, the potential link between the prolonged use of protective face masks and temporomandibular joint (TMJ) dysfunction or related discomfort is evident. Patients who present with a mask-related TMJ disorder showcase symptoms such as pain in the preauricular area, stiffness, articular noises, headaches, and pressure in the joint area. One of the examined studies states that the quality of life of patients with mask-related TMD is decreased. Many of the symptoms presented by the patients are also known to decrease the QoL by themselves.

All reviewed studies state that temporomandibular disorder symptoms deteriorate if a mask is worn for longer periods. Specifically, wearing a face mask for longer than 4–5 h a day may cause TMD symptoms in most cases.

The distinct type of mask seems to be of little to no relevance. All four studies show different results regarding whether cloth, surgical, or FFP masks are the healthiest.

The mask should be well fitted to be safe to the TMJ. Masks that do not fit well cause the wearer to perform repetitive movements to correct the mask’s fit. All studies address repetitive movements of the jaw as a crucial element in the development of TMDs.

Additionally, researchers must prioritize investigations into professions that entail prolonged mask use, such as surgeons, operating room staff, and pathologists. By doing so, we can implement improved work hygiene and safety measures to safeguard the well-being of these essential workers. We recommend further research to provoke more comfortable and less harmful solutions to mask structures, simultaneously improving the quality of life for workers who are using them daily. Mask producers could enhance the usefulness and user experience of their products by creating different sizes of masks and testing various materials from which masks could be created. For now, occupational safety and health measures should be taken. Such measures could mean decreasing the time of wearing masks to a minimum, taking breaks from wearing masks, changing the mask during the day, or using different types of masks.

The scarcity of research in this area is concerning, underscoring the need for further investigation to better understand its impact on individuals’ quality of life. Retrospective studies examining patients diagnosed with temporomandibular disorders (TMDs) during the COVID-19 pandemic could offer invaluable insights into this issue.

## Figures and Tables

**Table 1:** Outcome of checklist for prevalence studies as a part of JBI critical appraisal tools.

No	Question	Zuhour et al. [35]	Marques-Sule et al. [36]
1	Was the sample frame appropriate to address the target population?	yes	yes
2	Were study participants sampled in an appropriate way?	yes	yes
3	Was the sample size adequate?	unclear	yes
4	Were the study subjects and the setting described in detail?	yes	yes
5	Was the data analysis conducted with sufficient coverage of the identified sample?	yes	yes
6	Were valid methods used for the identification of the condition?	yes	yes
7	Was the condition measured in a standard, reliable way for all participants?	unclear	unclear
8	Was there appropriate statistical analysis?	yes	yes
9	Was the response rate adequate, and if not, was the low response rate managed appropriately?	yes	yes

**Table 2:** Outcome of checklist for cross-sectional studies as a part of JBI critical appraisal tools.

No	Question	D’Apuzzo et al. [37]	Carikci et al. [38]
1	Were the criteria for inclusion in the sample clearly defined?	no	yes
2	Were the study subjects and the setting described in detail?	yes	yes
3	Was the exposure measured in a valid and reliable way?	yes	yes
4	Were objective, standard criteria used for measurement of the condition?	yes	yes
5	Were confounding factors identified?	yes	yes
6	Were strategies to deal with confounding factors stated?	yes	yes
7	Were the outcomes measured in a valid and reliable way?	no	yes
8	Was appropriate statistical analysis used?	yes	yes

**Table 3:** Studies included in this review.

Author	Participants (Male/Female)	Country of Affiliation of Authors	Method	Aims	Main Symptoms
Zuhour et al. [35]	148 (excluded 42) -> in total 106 (39:67)	Turkey	Survey Questionnaire MRI	To investigate effects of repetitive jaw movements while wearing face masks on TMD	Pain of the TMJ, articular noises, joint tension
Marques-Sule et al. [36]	629 (excluded 87) -> in total 542(191:351)	Spain/Canada	Online Questionnaire	To assess presence, frequency and impact of headache, temporomandibular disorders, and quality of life (QoL)	TMJ discomfort
d’Apuzzo et al. [37]	665 (2 excluded) -> 663 (314:349)	Italy	Cross-sectional survey	Possible association of mask wearing with sings of TMD and orofacial pain	Preauricular pain
Carikci et al. [38]	909 (339:570)	Turkey	Cross-sectional survey Trigger Points of the TMJ on M. Temporalis and M. Masseter	To investigate the effects of long-term mask use on temporomandibular pain, headache, and fatigue during the COVID-19 pandemic period	Pain at TMJ, masseter and temporal muscle trigger points

**Table 4:** Symptoms reported in the study by Marques-Sule et al. [36].

No	Symptom	% of the Participants
1	Awake bruxism	42.8
2	Sleep bruxism	26.6
3	Chewing discomfort	7.2
4	TMJ pain	6.8

**Table 5:** Correlation between preauricular pain and mask use duration in the study by d’Apuzzo et al. [37].

	No (%)	Yes (%)
<4 h in total	41.1	31.5
4–8 h in total	30.4	36.8
>8 h in total	28.5	31.3
<4 h consecutive	60.5	49.3
4–8 h consecutive	30.0	38.8
>8 h consecutive	9.1	11.8

**Table 6:** Correlation between preauricular pain and talking in the study by d’Apuzzo et al. [37].

Talking	No Pain (%)	Stable/Increased Pain (%)	New Pain (%)
Never	89.7	41.3	55.5
Many hours	8.6	47.8	30.7
Always	1.3	10.9	13.1
Missing	0.4	0.0	0.7

**Table 7:** Characteristics of assessed groups in the study by Carikci et al. [38].

		Group 1	Group 2	Group 3
Mask type, n	Surgical	330	266	222
	Fabric	28	10	3
	N95	11	18	8
	Silver ion	2	4	1
	Other	2	2	2
Apparatus, n	Hook behind the ear	326	263	201
	Head–neck apparatus	47	37	35
Double mask, n	Yes	36	53	56
	Sometimes	114	120	102
	No	223	127	78
Daily mask change, n	None	110	46	21
	1	154	91	47
	2	78	107	70
	3	20	33	57
	>3	11	23	41

**Table 8:** Prevalence of bruxism in studies by Marques-Sule et al. [36] and Carikci et al. [38] (only studies that gathered data about this symptom).

Symptom	Marques-Sule et al. [36] (*N* = 542)	Carikci et al. [38] (*N* = 909)	In Total (*N* = 1451)
Bruxism	376	124	500 (34.5%)

**Table 9:** Prevalence of articular noise in studies by Zuhour et al. and d’Apuzzo et al. (only studies that gathered data about this symptom).

Symptom	Zuhour et al. [35] (*N* = 106)	d’Apuzzo et al. [37] (*N* = 663)	In Total (*N* = 769)
Articular noise	82	50	132 (17.2%)

**Table 10:** Prevalence of TMJ pain in all of the studies found.

Symptom	Zuhour et al. [35] (*N* = 106)	Marques-Sule et al. [36] (*N* = 542)	d’Apuzzo et al. [37] (*N* = 663)	Carikci et al. [38] (*N* = 909)	In Total (*N* = 2220)
TMJ pain	88	43	183	89	403 (18.2%)

**Table 11:** Correlation between durations of mask use and TMJ pain in the studies by d’Apuzzo et al. and Carikci et al. (these data could only be summarized from these studies).

Symptom	d’Apuzzo et al. [37] (*N* = 663)	Carikci et al. [38] (*N* = 909)	In Total (*N* = 1572)
TMJ pain in groups wearing masks for less than 4 h	44	24	68 (4.3%)
TMJ pain in groups wearing masks for more than 4 h	138	65	203 (12.9%)

**Table 12:** Occurrence of headache in the studies that researched it.

Symptom	Marques-Sule et al. [36] (*N* = 542)	d’Apuzzo et al. [37] (*N* = 663)	Carikci et al. [38] (*N* = 909)	In Total (*N* = 2114)
Headache	202	253	376	831 (39.3%)

## Supplementary Materials

The following supporting information can be downloaded at: https://www.mdpi.com/article/10.3390/medicina60091468/s1, Supplementary Materials S1–S5.
