# Supplementary material for: Potential Impacts of Prolonged Face Mask Use on Temporomandibular Joint Health as Neglected Lifestyle Repercussions of COVID-19 Pandemic—A Narrative Review
Source: Medicina (Kaunas). 2024 Sep 8;60(9):1468. doi: 10.3390/medicina60091468 (PMC11434408; doi:10.3390/medicina60091468)
Supplement: Supplementary file 1 [file medicina-60-01468-s001.zip › Supplementary material S1.pdf]

Database	Search strategy	Studies identified
PubMed	(("face mask" AND ("temporomandibular joint" OR TMJ))) AND (degeneration” OR “pathology” OR “injury” OR “influence” OR “pain” OR “discomfort” OR “stiffness” OR “TMD” OR “arthralgia”)	4
Google Scholar	(("face mask" AND ("temporomandibular joint" OR TMJ))) AND (“degeneration” OR “pathology” OR “injury” OR “influence” OR “pain” OR “discomfort” OR “stiffness” OR “TMD” OR “arthralgia”)	4
Embase	('face mask'/exp OR 'face mask') AND ('temporomandibular joint'/exp OR 'temporomandibular joint' OR tmj) AND ('degeneration'/exp OR 'degeneration' OR 'pathology'/exp OR 'pathology' OR 'injury'/exp OR 'injury' OR 'influence' OR 'pain'/exp OR 'pain' OR 'discomfort'/exp OR 'discomfort' OR 'stiffness'/exp OR 'stiffness' OR 'tmd' OR 'arthralgia'/exp OR 'arthralgia')	5
Ebsco	(("face mask" AND ("temporomandibular joint" OR TMJ))) AND (“degeneration” OR “pathology” OR “injury” OR “influence” OR “pain” OR “discomfort” OR “stiffness” OR “TMD” OR “arthralgia”)	4
Web of Science	(("face mask" AND ("temporomandibular joint" OR TMJ))) AND (degeneration” OR “pathology” OR “injury” OR “influence” OR “pain” OR “discomfort” OR “stiffness” OR “TMD” OR “arthralgia”)	4
Total		21
