# Supplementary material for: Potential Impacts of Prolonged Face Mask Use on Temporomandibular Joint Health as Neglected Lifestyle Repercussions of COVID-19 Pandemic—A Narrative Review
Source: Medicina (Kaunas). 2024 Sep 8;60(9):1468. doi: 10.3390/medicina60091468 (PMC11434408; doi:10.3390/medicina60091468)
Supplement: Supplementary file 1 [file medicina-60-01468-s001.zip › Supplementary material S2.pdf]

JBI CRITICAL APPRAISAL CHECKLIST FOR STUDIES REPORTING PREVALENCE DATA

Reviewer_____Martyna Kowalczyk___________________Date_____04.05.2024_______________

Author______Zuhour et al._______________________Year__2022_ Record Number_10.1055/s-0042-1743131

	Yes	No	Unclear	Not applicable
1. Was the sample frame appropriate to address the target population?	☒	☐	☐	☐
2. Were study participants sampled in an appropriate way?	☒	☐	☐	☐
3. Was the sample size adequate?	☐	☐	☒	☐
4. Were the study subjects and the setting described in detail?	☒	☐	☐	☐
5. Was the data analysis conducted with sufficient coverage of the identified sample?	☒	☐	☐	☐
6. Were valid methods used for the identification of the condition?	☒	☐	☐	☐
7. Was the condition measured in a standard, reliable way for all participants?	☐	☐	☒	☐
8. Was there appropriate statistical analysis?	☒	☐	☐	☐
9. Was the response rate adequate, and if not, was the low response rate managed appropriately?	☒	☐	☐	☐

Overall appraisal: Include ☒ Exclude ☐ Seek further info ☐

Comments (Including reason for exclusion)

Study by Zuhour et al. turned out to be appropriate for our research.
