# Supplementary material for: Potential Impacts of Prolonged Face Mask Use on Temporomandibular Joint Health as Neglected Lifestyle Repercussions of COVID-19 Pandemic—A Narrative Review
Source: Medicina (Kaunas). 2024 Sep 8;60(9):1468. doi: 10.3390/medicina60091468 (PMC11434408; doi:10.3390/medicina60091468)
Supplement: Supplementary file 1 [file medicina-60-01468-s001.zip › Supplementary material S5.pdf]

JBI CRITICAL APPRAISAL CHECKLIST FOR ANALYTICAL CROSS SECTIONAL STUDIES

Reviewer_____Martyna Kowalczyk___________Date______04.05.2024___________________

Author____Carikci et al.___________Year__2022____Record Number____10.1080/08869634.2022.2126916

	Yes	No	Unclear	Not applicable
1. Were the criteria for inclusion in the sample clearly defined?	☒	☐	☐	☐
2. Were the study subjects and the setting described in detail?	☒	☐	☐	☐
3. Was the exposure measured in a valid and reliable way?	☒	☐	☐	☐
4. Were objective, standard criteria used for measurement of the condition?	☒	☐	☐	☐
5. Were confounding factors identified?	☒	☐	☐	☐
6. Were strategies to deal with confounding factors stated?	☒	☐	☐	☐
7. Were the outcomes measured in a valid and reliable way?	☒	☐	☐	☐
8. Was appropriate statistical analysis used?	☒	☐	☐	☐

Overall appraisal: Include ☒ Exclude ☐ Seek further info ☐

Comments (Including reason for exclusion)

Study by Carikci et al. was appropriate to use in our review.
